# Mapping brain networks in MPS I mice and their restoration following gene therapy

**DOI:** 10.1038/s41598-023-39939-0

**Published:** 2023-08-05

**Authors:** Wei Zhu, Li Ou, Lin Zhang, Isaac H. Clark, Ying Zhang, Xiao-Hong Zhu, Chester B. Whitley, Perry B. Hackett, Walter C. Low, Wei Chen

**Affiliations:** 1 Center for Magnetic Resonance Research, University of Minnesota, Minneapolis, MN 55455 USA; 2 Department of Radiology, University of Minnesota, Minneapolis, MN 55455 USA; 3 Department of Pediatrics, University of Minnesota, Minneapolis, MN 55455 USA; 4 Present Address: Genemagic Biosciences, Media, PA 19063 USA; 5 Division of Biostatistics, University of Minnesota, Minneapolis, MN 55455 USA; 6 Biomedical Engineering Graduate Program, University of Minnesota, Minneapolis, MN 55455 USA; 7 Minnesota Supercomputing Institute, University of Minnesota, Minneapolis, MN 55455 USA; 8 Department of Genetics, Cell Biology Development, University of Minnesota, Minneapolis, MN 55455 USA; 9 Stem Cell Institute, University of Minnesota, Minneapolis, MN 55455 USA; 10 Department of Neurosurgery, University of Minnesota, Minneapolis, MN 55455 USA; 11 Graduate Program in Neuroscience, University of Minnesota, Minneapolis, MN 55455 USA

**Keywords:** Biotechnology, Neuroscience, Biomarkers, Diseases, Nanoscience and technology

## Abstract

Mucopolysaccharidosis type I (MPS I) is an inherited lysosomal disorder that causes syndromes characterized by physiological dysfunction in many organs and tissues. Despite the recognizable morphological and behavioral deficits associated with MPS I, neither the underlying alterations in functional neural connectivity nor its restoration following gene therapy have been shown. By employing high-resolution resting-state fMRI (rs-fMRI), we found significant reductions in functional neural connectivity in the limbic areas of the brain that play key roles in learning and memory in MPS I mice, and that adeno-associated virus (AAV)-mediated gene therapy can reestablish most brain connectivity. Using logistic regression in MPS I and treated animals, we identified functional networks with the most alterations. The rs-fMRI and statistical methods should be translatable into clinical evaluation of humans with neurological disorders.

## Introduction

Lysosomal disorders (LDs) in humans cause syndromes characterized by physiological impairments and dysfunction in many organs and tissues including the central nervous system (CNS) where they can result in severe cognitive deficiencies^1^. Among LDs, mucopolysaccharidosis type I (MPS I) results from any of several mutations in the gene that encodes α-l-iduronidase (IDUA), which when completely deficient is known clinically as Hurler Syndrome. IDUA deficiency leads to an altered metabolism and lysosomal accumulation of glycoaminoglycans (GAGs) such as heparan sulfate and dermatan sulfate that may play key roles in impairing synapse formation within the CNS^2,3^. The undegraded GAGs also cause morphological changes, with for example significant decreases in the volume of the thalamus and pallidum (striatum), but no significant shrinkage of the hippocampal formation^4^. Although morphological and behavioral deficits are well characterized in MPS I^5–7^, the underlying alterations in functional neural connectivity are unknown in humans and animal models used to develop therapeutic approaches. Evaluation of the efficacy of prospective treatments in animals are often superficial due to testing that involves physical activities such as negotiating mazes and spatial memory. For instance, in animal models in which gene therapy can provide IDUA to reduce GAG levels in the brain and significantly improve spatial navigation and reference memory^8^, the extent to which brain functions and associated neural connectivity can be restored has not been assessed. Current therapies are some of the most expensive in medicine even though their efficacy is not clear and evaluating new approaches using animal models is compromised by the lack of definitive tests of neural restoration^9^.

Despite the demand of functional evaluation of the neurological involvement of MPS diseases before and after treatment, few studies have mapped and visualized functional neural connectivity across the brain^10^. To bridge this gap, we employed high-resolution resting-state functional MRI (rs-fMRI)^11^ as a safe, noninvasive, and whole-brain activity imaging tool for MPS diagnosis and post-treatment evaluation. Rs-fMRI investigates the spontaneous blood oxygenation level dependent (BOLD) fluctuation^12^ and its temporal correlation among different brain regions in the absence of stimulations or cognitive tasks. As temporal correlation of spontaneous BOLD fluctuation is assumed to be closely associated with neural synchronization^13^, rs-fMRI serves as an excellent surrogate of intrinsic brain activity from which spontaneous functional connectivity involving multiple brain regions can be identified to generate multiple resting state networks (RSNs)^14–20^. Clinically, rs-fMRI has identified a large number of RSNs that underlie various normal and pathophysiological behavioral states such as Alzheimer’s disease^21,22^, major depression^23,24^, and schizophrenia^25^. These studies demonstrate that deficits in neural network and interconnectivity are the underlying basis for specific neurological disorders. Herein, we hypothesized that learning and memory, and spatial navigation deficits observed in MPS I mice are mediated by alterations in limbic network connectivity such as the hippocampal formation, anterior cingulate cortex, retrosplenial cortex, and thalamus, and the rs-fMRI should provide a sensitive imaging tool to assess the impaired RSNs in MPS I brain and restored RSNs after gene treatment.

To test the hypotheses, we employed high-resolution rs-fMRI at ultrahigh field (9.4 Tesla) to evaluate and identify neural networks that were disrupted within the brain of MPS I mice and the extent to which brain functions and connectivity were restored following the gene therapy. We found significant reductions in functional neural connectivity in limbic areas of the MPS I brain that play key roles in learning and memory. Following adeno-associated virus (AAV)-mediated gene therapy to MPS I mice, we observed significant increases in IDUA levels within the brain and reinstatement of functional connectivity between limbic regions. These findings suggest that neural networks that involve the hippocampus, thalamus, and anterior cingulate area can be used to evaluate severity of neurological deficits in MPS I, with the anterior cingulate cortex network exceeding 99% of accuracy for prediction of LD. Our findings are the first to demonstrate the normalization of brain connectivity in a neurological disorder that can be treated by gene therapy and provide a new standard for evaluation of gene therapies for correction of neural network activities within the brain that underlie cognitive performance.

## Results

### Generalized evaluation and prediction pipeline

**Figure 1:**
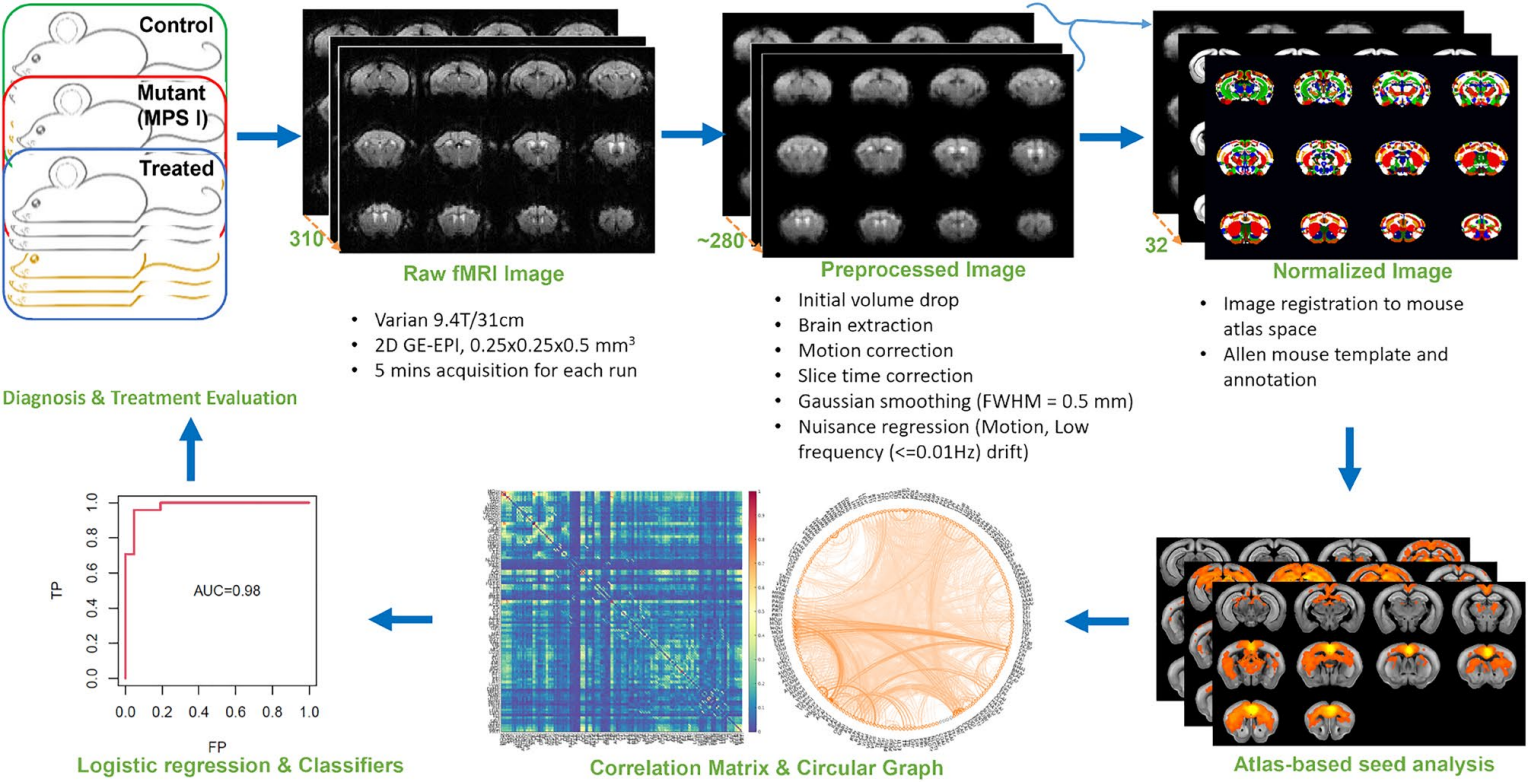
Resting state functional magnetic resonance imaging data analysis pipeline. Three cohorts of mice were scanned in the 9.4 Tesla Varian MRI scanner with the bore size of 31 cm. FMRI raw data were acquired using the 2D gradient echo-based echo planer imaging (GE-EPI) sequence with a nominal spatial resolution of 0.25 × 0.25 × 0.5 mm^3^ and temporal resolution of 1 s. Three to five fMRI runs were acquired for each mouse with 5 min acquisition for each run. Raw fMRI images were preprocessed using the standard fMRI preprocessing pipeline and thereafter normalized to Allen mouse atlas. By using labeled brain regions as seedings, Pearson’s correlations were calculated between the seeding region and all fMRI voxels to generate RSNs. The region-to-region correlations were also calculated to derive the correlation matrix, which can be visualized using circular graph. Logistic regression was then conducted on either the correlation matrices or the RSNs for each cohort to generate spatial and ROI (region of interest) classifiers that were used to predict the three cohorts.

Given the cognitive deficits associated with Hurler syndrome^1^, determining functional connectivity between areas of the brain is more important than their relative morphological changes. Hence, we employed rs-fMRI to reveal patterns of connectivity within the entire brain in adult (8-month) normal, MPS I mutant mice, and MPS I mice treated with gene therapy at 4–6 weeks using adeno-associated virus type 8 carrying an IDUA expression cassette (AAV8-IDUA). We built a processing pipeline from data acquisition to functional network classifier modeling for evaluation and prediction (Fig. 1). Specifically, high-resolution BOLD-based rs-fMRI data acquired at 9.4 Tesla were preprocessed to remove motion and low frequency drift^26^. Taking advantage of the mouse brain atlas and parcellation from the Allen Institute^27^, we normalized each mouse to the atlas and performed seed analysis to generate RSNs. Each brain region, from a total of 235, can be designated as a “seed” or reference point with which to compare the temporal changes and coherences of BOLD signals to other regions. Areas of brain that exhibit highly synchronized changes in their BOLD signals were deemed functionally connected. Region-to-region correlation was calculated to obtain the adjacency matrix that can be visualized by the circular graph. Logistic regression was used to find spatial classifiers for diagnostic and evaluative purposes.

### Connectivity of brain domains in mice revealed by rs-fMRI

**Figure 2:**
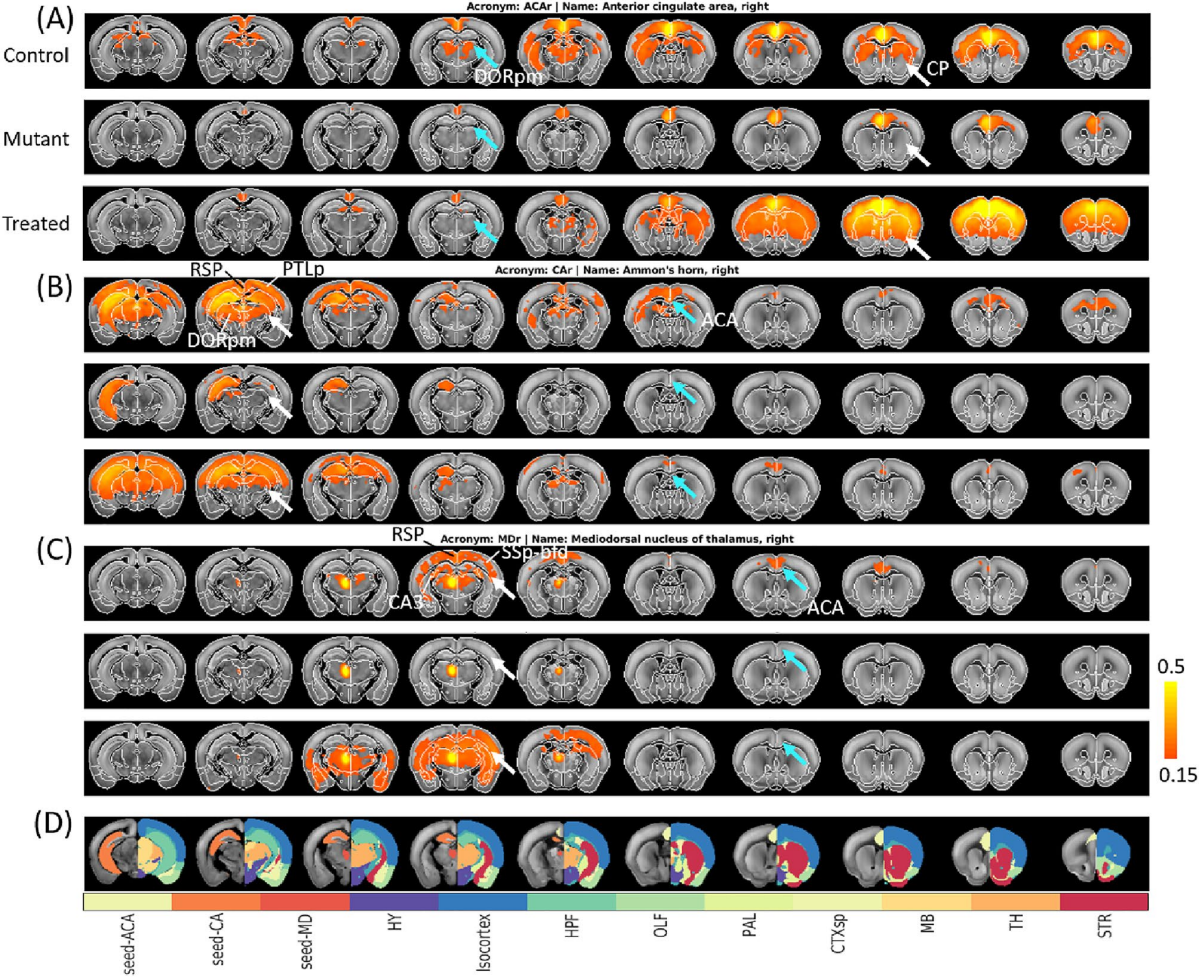
Comparison of resting-sate fMRI brain connectomes. Three example seed reference areas: (**A**) anterior cingulate area, right-hemispheric brain (ACAr), (**B**) Ammon's horn, right-hemispheric brain (CAr), and (**C**) mediodorsal nucleus of thalamus, right-hemispheric brain (MDr) for control (top), mutant (MPS I, middle), and treated (bottom) cohort, respectively, with seeding areas and other main brain areas shown in different colors in the atlas maps (**D**) for reference. The resting state networks were displayed by setting the cross correlation coefficient threshold at 0.15 with p-value < 0.05 (uncorrected). White arrows are example brain regions whose functional connectivity to other regions recovered after gene therapy; cyan arrows are regions with unrestored functional connectivity even after gene therapy. *DORpm* dorsal thalamus, polymodal association cortex related, *CP* caudoputamen, *RSP* retrosplenial area, *PTLp* posterior parietal association areas, *SSp* primary somatosensory area, *HY* hypothalamus, *HPF* hippocampal formation, *OLF* olfactory areas, *PAL* pallidum, *CTXsp* cortical subplate, *MB* midbrain, *TH* thalamus, *STR* striatum. The right-hemispheric brain is shown on the left side of the brain images.

We first determined the functional connectivity of each neural region defined in the Allen mouse brain atlas in wild-type mice to establish the best parameters for obtaining RSNs. Using the same protocol, we compared the RSNs in MPS I mice and MPS I mice following IDUA gene therapy. Three representative RSNs with seeding at the anterior cingulate area (ACA), Ammon’s horn (CA, hippocampus), mediodorsal nucleus of thalamus (MD) in the right hemisphere are shown, respectively, in Fig. 2. The RSNs were displayed by setting a cross correlation coefficient threshold at 0.15 with p-value < 0.05 (uncorrected).

In general, RSNs in wild-type mouse brains revealed extensive functional connectivity across the brain, including both hemispheres (first rows in Fig. 2A–C). In contrast, the MPS I brain exhibited a much weakened and altered connectome involving various cortical and subcortical regions critical for learning, memory, and sensorimotor behavior (second rows in Fig. 2A–C). Specifically, using the ACA in wild-type mice as a reference point revealed functional connections to the motor cortex and caudoputamen of the dorsal striatum (first row in Fig. 2A, with reference to Fig. 2D). More caudally, the retrosplenial cortex, thalamus, and hippocampus showed functional connectivity with the anterior cingulate. These networks form so-called default mode networks (DMNs). In sharp contrast, MPS I mice exhibited a profound loss of functional connectivity in DMNs. The functional connectivity loss involving ACA and striatum was more severe for MPS I female mice than male mice with p-value < 0.01 (second row in Figs. S1A and S2A). In MPS I mice that received IDUA gene therapy, functional connections between the anterior cingulate and the motor cortex, dorsal striatum, and hippocampus were restored. As in the hippocampus and thalamus, this pattern of functional innervation was even more pronounced in MPS I female mice that received gene therapy with p-value < 0.01 (third row in Figs. S1A and S2A). However, gene therapy did not bring back all connections, especially those distant to the seeding areas. For instance, the communication between ACA and dorsal thalamus that appeared in normal mice was still absent in the treated mice (cyan arrows in Fig. 2A).

RSNs of the Ammon’s horn (CA) revealed extensive functional connectivity with various limbic areas of the brain and, in particular, the retrosplenial cortex, anterior cingulate area and thalamus (Fig. 2B). In addition, there was extensive connectivity with the contralateral CA region of the hippocampus. In contrast to the wild-type mice, the MPS I brain exhibited dramatic decreases in hippocampal connectivity with other limbic areas. Specifically, functional connectivity between the CA region of the hippocampus and the retrosplenial cortex, anterior cingulate area, entorhinal cortex and contralateral hippocampus was essentially absent. Female MPS I mice even lost the ipsilateral connections of CA region to other nearby regions (second row in Figs. S1B and S2B). For gene therapy treated MPS I mice with enhanced IDUA levels, we found reinstatement of CA hippocampal connectivity in both sexes but with greater functional connectivity recovery in female mice (third row in Figs. S1B and S2B). Moreover, in female mice functional connectivity between the hippocampus on the right side of the brain and the dorsal striatum bilaterally was greater than that observed in wild-type mice.

Similarly, when seeding the mediodorsal nucleus of thalamus, we observed a high correlation of spontaneous neural activity in normal brains with the contralateral nucleus, ipsilateral and contralateral hippocampus (CA3), bilateral retrosplenial cortex, bilateral motor and somatosensory cortices, and bilateral anterior cingulate cortices (Fig. 2C). In MPS I mice, functional connectivity with the contralateral thalamus as well as other brain areas was greatly diminished for both male and female mice. Following gene therapy, MPS I mice exhibited restored functional connectivity with the contralateral thalamus, hippocampus, somatosensory cortices, and cingulate cortices. The restoration of thalamic connectivity was far more robust in female mice (third row in Figs. S1C and S2C), and high levels of functional connectivity was also observed between the thalamus and contralateral thalamus, bilateral hippocampus, bilateral motor and somatosensory cortices, bilateral septal nuclei, and bilateral dorsal striatum. In addition, areas of the ventral striatum, and piriform cortex also exhibited extensive connectivity with the thalamus following gene therapy.

**Figure 3:**
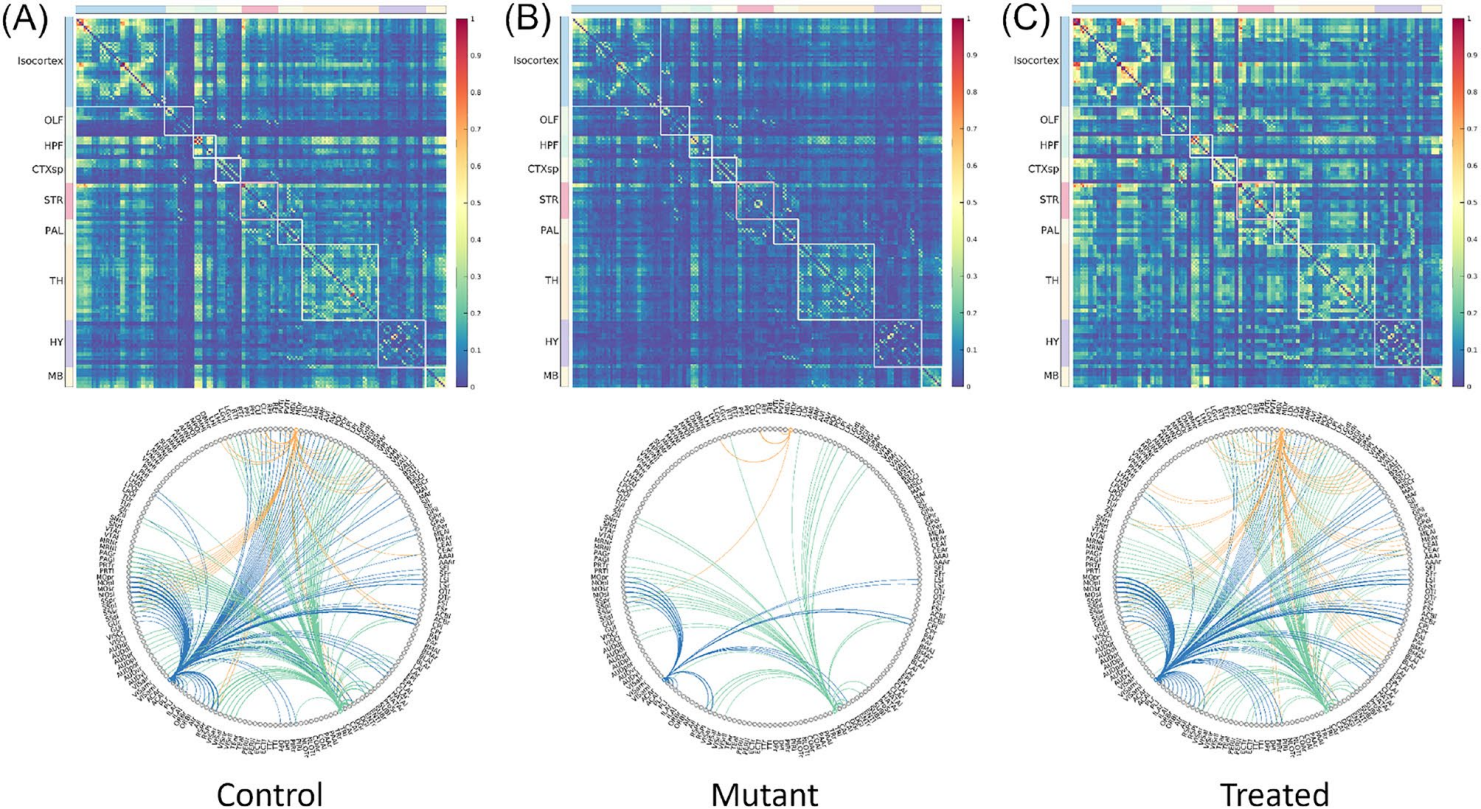
Correlation matrices and circular graphs for (**A**) control, (**B**) MPS I, and (**C**) MPS I-treated mice. The connectome graphs in the second row with the same seeding areas as those in Fig. 2A–C were color coded: blue-connectome with seeding at ACAr, green-connectome with seeding at CAr, and yellow-connectome with seeding at MDr. Brain connectome is sparse for mutant cohort whereas it recovers after treatment.

To provide a graphical comparative visualization of the functional brain connections between normal, MPS I, and MPS I-treated mice, connectivity matrices were generated using the correlation coefficients between different areas of the brain (top row, Fig. 3). Consistent with the RSNs, connectivity matrices of normal mice revealed a high degree of functional linkage in the whole brain. In contrast, MPS I mice exhibited a paucity of functional connections whereas MPS I mice that received AAV-mediated, IDUA gene therapy displayed marked restoration of functional connectivity.

To visualize differences in functional connectivity with the correlation coefficient strength larger than 0.2, connectograms were generated for the three representative seeding areas used to generate RSNs in Fig. 2 (bottom row in Fig. 3, ACA, blue; CA, green; MD, yellow). Connectograms represent the degree of functional connectivity from one specific area of the brain to its various targets for innervation. The connectogram for the ACA on both the left and right sides of the wild-type brain exhibited strong connectivity to the contralateral counterpart as well as robust bilateral connections to the hippocampus, thalamus, and striatum in wild-type mice. These functional connections were greatly diminished in MPS I mice, and mostly reinstated in MPS I mice following gene therapy. Similar trends were observed on the connectogram for the CA. After gene therapy, the recovered functional connections were not identical to the control, with stronger connections in areas spatially close to the CA (such as thalamus and RSP) and weaker or no connections in areas spatially farther from the CA (such as ACA). The connectivity recovery after gene treatment was even more pronounced in the connectogram for the MD in thalamus.

### Classification models based on logistic regression

To determine whether alterations in functional connectivity in specific neural networks could be a predictor for distinguishing between normal, MPS I, and treated MPS I mice, we used the bootstrap aggregating algorithm^28^ to build a classification model with multi-resolution correlation maps. Classification models were obtained for each bootstrap sample using the logistic LASSO regression^29^ and the final ensemble classifier was obtained by averaging 1000 classification models. Ensemble classifiers associated with the ACA, CA, and MD networks were identified. The predictive power of each ensemble classifier and the one combining all the ensemble classifiers was then evaluated using receiver operating curve (ROC) analysis.

**Figure 4:**
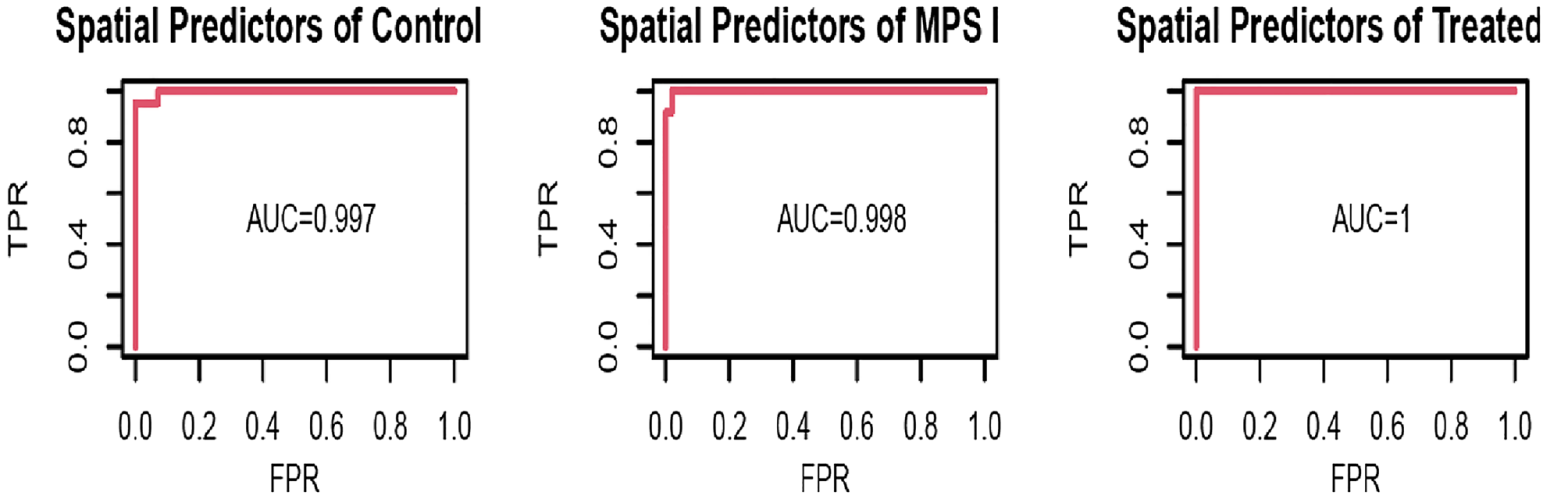
Receiver operating curve analysis of AUC accuracy for predicting wild-type, MPS I, and treated MPS I mice using a logistic regression model by combining connectivity of ACA, CA, and MD with other brain regions with significant predictive power. The combined network biomarker ensemble leads to AUC values of 99.7%, 99.8%, and 100% of accuracy for distinguishing three groups, respectively.

Significant functional networks between the seeding area and the predictor areas (p-value < 0.001) identified from the ensemble classifiers are listed in Table 1. Significant networks in first ensemble classifiers were the connectivity of the ACA region of the brain with sensory cortex, parasubiculum area, magnocellular nucleus, nucleus of reuniens, and lateral septal nucleus. This ensemble network alone produced an AUC value of 98% for distinguishing between control, MPS I, and treated groups (Fig. S3). The significant ensemble classifier that related to CA region includes network alterations between subregions in CA, perirhinal area, and pretectal region. ROC analysis of these networks alone suggests rs-fMRI connectivity has a predictive biomarker accuracy of 94%. Another ensemble classifier consisted of functional connections between thalamus and perirhinal area, piriform-amygdalar area, basomedial amygdalar nucleus, and subiculum. This ensemble network resulted in a biomarker with a 97% AUC diagnostic accuracy value. By combining these classifiers, we obtained a single classification model to predict control, MPS I and treated individuals—the combined ensemble classifier has a 99.7% AUC diagnostic accuracy value (Fig. 4).

**Table 1:** Significant functional networks between the seeding area and the predictor areas (p-value < 0.001) identified from the ensemble classifiers using logistic regression.

Seeding area	Predictor acronym	Predictor full name	Functions
ACA	ACA	Anterior cingulate area	Autonomic functions (e.g., heart rate), attention allocation, decision-making
	MO, GU	Motor, gustatory, auditory, visual areas	Sensory information processing
	PAR	Parasubiculum area	Major projections to the superficial layers of the entorhinal cortex^30^, spatial navigation and head-directional information^31^
	MA	Magnocellular nucleus	Motor coordination^32^
	RE	Nucleus of reuniens	Memory and emotion^33^
	LS	Lateral septal nucleus	Motivation and reward, social behavior, memory formation and retention
CA	CA	Ammon’s horn	Memory and spatial cognition, approach-avoidance conflict processing
	PERI	Perirhinal area	Visual perception and memory^34^
	PRT	Pretectal region	Mediating behavioral responses to acute changes in ambient light
MD	PERI	Perirhinal area	Visual perception and memory^34^
	PAA	Piriform-amygdalar area	Emotional and memory processing^35^
	BMA	Basomedial amygdalar nucleus	Fear response and pain memory
	SUB	Subiculum	Working memory^36^, spatial relations^37^

### Evaluation of gene therapy to restore neural connectivity in brain of MPS I mice

Previously, we showed that AAV8-mediated, IDUA gene transfer into the ventricles of MPS I mice resulted in the expression of high levels of IDUA in neurons in the hippocampal formation that was correlated with the restoration of spatial navigation in a water maze task^38^. Expressing IDUA from the endogenous albumin promoter led to *supra*-physiological levels of serum IDUA activity that were 100-fold greater than normal, and brain IDUA twofold more than normal along with a corresponding decrease in neuropathology in the treated mice^8^. High-dose systemic enzyme replacement therapy with IDUA in MPS I mice can also significantly increase IDUA activity in the brain and reduce GAG levels in the cortex and cerebellum that are associated with significant improvements in water T-maze spatial navigation and reference memory tests^39^.

**Figure 5:**
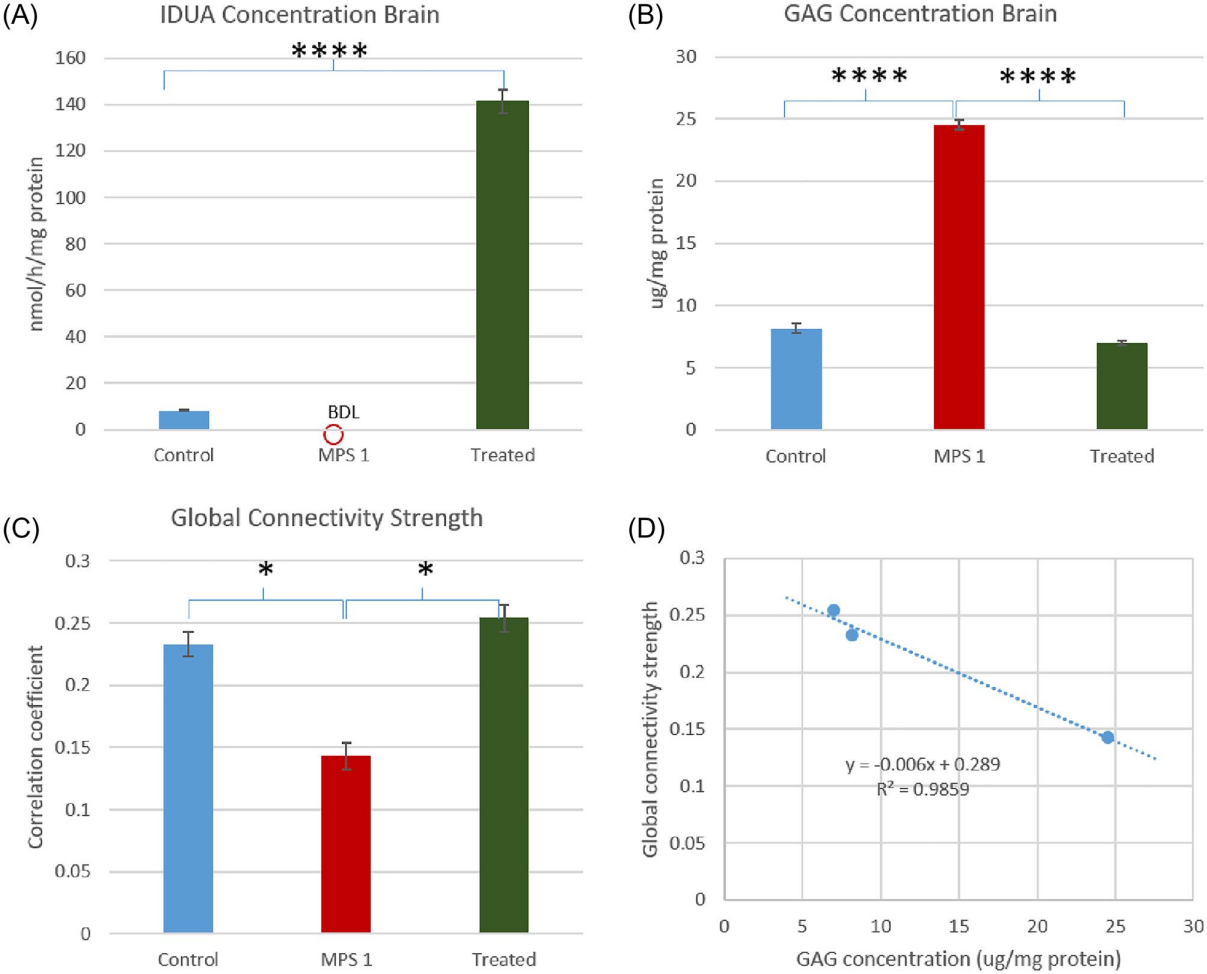
Comparison of brain concentrations of (**A**) IDUA, (**B**) GAG, and (**C**) global functional connectivity strength in wild-type, MPS I and treated MPS I mice, and (**D**) correlation between GAG concentration and global connectivity strength. According to the results, GAG level was inversely correlated with global connectivity strength with R^2^ = 0.99. In MPS I mice, IDUA was below detection level (BDL), leading to about threefold higher GAG level. Note that SEM error bars were used in the histograms to provide visual information regarding the variability of the estimates.

After the imaging measurements, we harvested the brains for assays of IDUA enzyme and GAG levels. In normal mice, the average whole-brain IDUA was 8.3 ± 0.2 nmol/h/mg protein (mean ± SEM) compared to below detection level (BDL) in MPS I mice (Fig. 5A). In contrast, treated male and female MPS I mice exhibited an average brain IDUA level of 141.3 ± 4.9 nmol/h/mg protein. Due to the large variance differences in IDUA levels among the three groups, nonparametric Kruskal–Wallis test was conducted to check the group difference: Chi-squared statistic = 23.2 with group degree of freedom (DOF) of 2 and residual DOF of 23, p-value = 9.25E−6; the p-value for all pairwise comparison is 2.2E−4. We also assessed the levels of GAGs within the brain (Fig. 5B); the average GAG level was 8.2 ± 0.4 μg/mg protein in normal mice and elevated to 24.5 ± 0.4 μg/mg protein in MPS I mice (p = 1.4E−10). In both male and female treated MPS I mice, the average GAG level dropped below normal to 6.9 ± 0.2 μg/mg protein (p = 6.5E−11 vs. MPS I mice). The statistics of one-way ANOVA in GAG group comparison were: F-value = 93.44 with group DOF of 2 and residual DOF of 23, p-value = 9.10E−12. The restored high IDUA level and consequential lowered GAG level following systemic gene therapy were consistent with restored functional connections (Fig. 5C). The global functional connectivity strength, calculated as the average of correlation coefficients (larger than 0.15) over all brain regions, was 0.23 ± 0.01 in normal mice, dropped to 0.14 ± 0.01 in MPS I mice, and raised to 0.25 ± 0.01 in treated MPS I mice (Fig. 3). The statistics of one-way ANOVA in group comparison were: F-value = 4.24 with group DOF of 2 and residual DOF of 17, p-value = 0.03. Further, we observed a large negative correlation between the GAG concentration and the global functional connectivity strength with R^2^ = 0.99. A positive association trend between IDUA level and global connectivity strength was also observed but the correlation is nonlinear likely due to the low IDUA concentration level in the MPS I mouse brain and saturated IDUA enzymes in the treated brains.

## Discussion

### Brain connectivity revealed by rs-fMRI supports conjectures on neural activities and neural networks underlying spatial memory and navigation

The results presented here demonstrate large deficits in the functional neural networks of MPS I mice that are associated with behavioral dysfunction stemming from mutations in the IDUA gene. Selective combinations of network interactions among limbic areas of the brain identified by logistic regression provide sensitive network biomarkers that can accurately distinguish between normal, MPS I, and effectively treated mice (Fig. 4). IDUA enzyme deficiency leads to accumulation of heparan sulfate and dermatan sulfate in the lysosomal compartment of cells, resulting in cellular dysfunction. The inability to metabolize glycosaminoglycans renders neural cells incapable of carrying out key roles in synapse formation during brain development by regulating axon interactions with signaling molecules such as Semaphorin 5^40^ and Ephrin A3^41^ for axon filopodia attraction, and Slit proteins^42^ for axonal repulsion. The affected synapse formation causes diminished neural connectivity, which is supported by our rs-fMRI results (Figs. 2, 3, and 5). Heparan sulfate proteoglycan (HSPG) subfamilies are also involved in the formation of synaptic connections. HSPG agrin is secreted by presynaptic terminals, and functions as a synaptic organizer^43,44^ when secreted within the presynaptic terminals and localized in the synaptic cleft to instruct postsynaptic development. The ability of HSPGs to sculpt synaptic development requires their association with specific binding partners. For example, EphB2 binding leads to the phosphorylation of syndecan 2 (SDC2) that initiates spine clustering and triggering of morphogenesis for dendritic spine development^3^. In cultured hippocampal neurons, overexpression of SDC2 results in the acceleration of mature dendritic spines^2^.

The expression pattern of HSPGs exhibits cell-type specificity that may contribute to the specificity of functional connectivity^45–48^. These studies underscored the importance of HSPG for axonal fiber innervation and cell-specific synapse formation. Alterations in the formation of appropriate synapses between neurons in the hippocampal formation and other areas of the limbic system may therefore represent the underlying structural and functional basis of deficits in brain connectivity that are observed in MPS I mice and, by extension, in other lysosomal enzyme deficiency disorders. Our demonstration of reversal of deficits in synaptic connectivity due to alterations in HSPG function in MPS I strongly supports further development of gene and cell therapies to introduce missing enzyme activity in other LDs and thereby restore neurological function by normalizing the brain connectome.

In designating the hippocampus on one side of the MPS I brain as a reference point, we found a complete lack of connectivity to the contralateral hippocampus and a loss of functional connectivity between the hippocampus and the retrosplenial cortex, anterior cingulate cortex, and entorhinal cortex compared with normal brains. The entorhinal cortex provides important spatial information to the hippocampal formation regarding “where” the animal is within its spatial environment, while the lateral entorhinal cortex provides information about “what” is contained within the spatial location^49^. The visual cues within the spatial environment enable the hippocampus to generate cognitive maps, which are represented as “place fields” as seen by the discharge of action potentials pyramidal neurons in the CA1 region of the hippocampus^50^. We hypothesize that the functional disconnection between the entorhinal cortex and the hippocampal dentate gyrus seen in our rs-fMRI studies most likely impedes the formation of the cognitive maps needed for the MPS I mice to perform the water maze task.

The loss of functional connectivity between the hippocampal formation and the retrosplenial cortex in MPS I mice would also have important consequences in spatial navigation performance. Neuroanatomical studies indicate that the hippocampal formation and the retrosplenial cortex are reciprocally connected^51^. The output from the hippocampus to the retrosplenial cortex originates in the CA1 and subicular regions of the hippocampal formation and project to retrosplenial granular A cortex (RGA). Behavioral studies indicate that head orientation is mediated by the retrosplenial cortex^52,53^ and lesions result in spatial memory deficits^54,55^. Importantly, our rs-fMRI brain connectivity results are consistent with the electrophysiological recordings, which reveal high coherence of theta activity between the two areas of the brain^56^ as well as gamma activity^57^.

The functional connectivity between the hippocampal formation and the thalamus is well documented^37,58,59^. So-called head direction cells are thought to encode primary information regarding spatial orientation within an environment. As we observed in the MPS I mice using rs-fMRI, the decrease in functional connectivity between the anterior thalamus and the hippocampal formation as well as the retrosplenial cortex directly supports conjectures of their poor performance in spatial navigation tasks. Likewise, the anterior cingulate cortex is highly interconnected with other structures of the limbic system. Neuroanatomical tracing studies document connections between the hippocampus^60,61^ and retrosplenial cortex^62^ in support of our rs-fMRI findings. A recent behavioral study demonstrates that the anterior cingulate cortex plays a critical role in remote spatial memory^63^. Our findings of decreased functional connectivity of the anterior cingulate cortex with the hippocampus in MPS I mice suggest that these animals would be unable to recall remote spatial memories; this hypothesis needs to be tested.

### Systemic gene therapy for MPS can enhance brain connectivity

In MPS I mice that received AAV8-mediated liver gene therapy to elevate systemic levels of IDUA, we observed marked enhancements in their brain connectome to the point where it resembled normal mice. Specifically, the hippocampal formation exhibited functional connectivity with the contralateral hippocampus, anterior thalamus, retrosplenial cortex, and anterior cingulate cortex. As described above, these regions of the limbic system are critical for spatial navigation and memory. The reinstatement of their functional inter-connectivity as observed with rs-fMRI provides clear evidence, at the level of neural networks, that high levels of systemic IDUA can restore neurological connectivity. However, the connectograms (Fig. 3) may not display all the regions in the brain with significant differences in correlation because the classification model neither requires all regions nor even symmetry between hemispheres to make a prediction. Among the brain regions used for classification, we found that in general MPS1 mice have lower correlations between the identified regions than controls. This makes sense as these regions are responsible for deficits in MPS I mice such as the right auditory areas linked to hearing loss^64^ and the entorhinal areas associated with spatial memory deficits^38^. In contrast, correlations between some regions were higher in MPS I mice compared to controls, including the right primary somatosensory and amygdala areas. These higher correlations may suggest subjects with MPS I would experience greater touch sensitivity as well as emotional responses; nevertheless, this hypothesis requires further investigation.

Interestingly, this study clearly indicates that the hippocampus serves as a central hub in many RSNs of CNS, and its functional connectivity with other brain regions was largely impaired in the MSP I mice, but, strikingly restored by gene treatment despite the lack of obvious morphological change and shrinkage of the hippocampal formation^4^. It supports our hypothesis that the rs-fMRI provides sensitive and early imaging biomarkers to assess the impaired RSNs in MPS I brain and restored RSNs after gene treatment. The overall results and findings from this study have significant clinical relevance for monitoring and treating patients with MPS I. The various mutations of the IDUA gene can result in a range of neurological severity. Characterizing and monitoring the brain connectome for MPS I patients can therefore be used to properly diagnose the pathological brain connectivity status of each patient, and monitor their responses to current enzyme replacement therapy and hematopoietic stem cell transplantation therapy. In this perspective, the rs-fMRI approach along with the logistic regression model as described in this work can be fully translated to the clinical setting. Importantly, the rs-fMRI based brain connectome can also be used to validate the efficacy of MPS 1 gene therapy in FDA-approved clinical trials and subsequently to monitor individual patient responses to this new therapy.

In sum, this study demonstrates for the first time that rs-fMRI supports noninvasive imaging to identify disrupted neural networks associated with neurological disorders resulting from genetic mutation and, more importantly, to evaluate the restoration of these networks following gene therapy. The same imaging approach should be readily translatable into clinical evaluation of humans with other neurological disorders and outcomes of gene therapy or other treatment.

## Methods

All experimental procedures carried out in this study were approved by the Institutional Animal Care and Use Committee (IACUC) of the University of Minnesota. All animal care and handling procedures were in accordance with relevant guidelines and regulations. All methods reported in this study were in compliance with the ARRIVE guidelines.

### Animals and AAV gene delivery

MPS I knockout mice (Idua^−/−^), a kind gift from Dr. Elizabeth Neufeld (University of California, Los Angeles [UCLA]), were maintained as a colony at the University of Minnesota. Mice were generated by inserting neomycin resistance gene into exon 6 of the 14-exon IDUA gene on the C57BL/6 background. MPS I mice (Idua^−/−^) and heterozygotes (Idua^−/+^) were genotyped by PCR. All MPS I mice (n = 8, 4–6 weeks old) were treated as adults through tail vein injections of two AAV8 vectors (5 × 10^9^ vg/g AAV8-SaCas9 and 3 × 10^10^ vg/g AAV8-IDUA-gRNA). To evaluate IDUA and GAG levels before and after gene treatment, MPS I mice with gene therapy (n = 8), wild-type control mice (n = 9), and untreated MPS I mice (n = 9) were examined for comparison at the same age of 8-month-old. All mice were from the same genetic background so as to control genetic noise in the experiment design.

### Gene construct design and AAV production

The gRNAs targeting the mouse albumin locus were designed and confirmed in embryonic fibroblast cells as previously described^65^. AAV-IDUA-gRNA and AAV-SaCas9 were packaged into AAV8 vectors at the Children’s Hospital of Philadelphia Research Vector Core. The quality of AAV vectors was verified by SDS-PAGE and silver staining. The core follows good laboratory practice (GLP) guidelines.

### Resting-state fMRI

MPS I mice (n = 6, 3 males and 3 females), gene treated MPS I mice (n = 8, 5 males and 3 females), and wild-type control mice (n = 6, 3 males and 3 females) with the same age (8-month-old) and similar body weights were scanned under a protocol approved by the IACUC at the University of Minnesota. All animals were inducted with 5% isoflurane mixed in O_2_:N_2_O (30:70) gas and anesthetized with 1.6% isoflurane during the preparation. When animal respiration rate became stable at 80–120 per minutes, a bolus of dexmedetomidine of 0.3 mg/kg in sterile physiological saline was injected IP. After 3 min, isoflurane was reduced continuously by 0.2% every two minutes and a continuous infusion of dexmedetomidine of 0.6 mg/kg/h started when isoflurane was reduced to 0.4%. Once the respiration rate was stable at a rate of > 140 per minutes, MRI data acquisition started. The physiology of the animals was monitored and well controlled throughout the study.

The MRI experiments were performed on a 9.4 T/31 cm animal scanner (Varian/VNMRJ, California, USA) using a single loop (1.5 cm diameter) proton radiofrequency surface coil. T_2_ weighted anatomical images were acquired with repetition/echo time (TR/TE) = 4000/10 ms; matrix = 256 × 128; field of view (FOV) = 2.4 × 1.2 cm; 12 slices with a thickness of 0.5 mm. Gradient echo (GE)-echo planar imaging (EPI) based rs-fMRI images were obtained with TR/TE = 1000/20 ms; matrix = 96 × 48; FOV = 2.4 × 1.2 cm; 12 slices with a thickness of 0.5 mm. For each mouse, two to five rs-fMRI datasets with 310 volumes were obtained with fat and muscle saturated. These rs-fMRI data were preprocessed following the standard pipeline programmed in MATLAB (The Mathworks Inc., Natick, MA) to generate 235 RSNs using seed analysis where each seed was a brain region outlined based on the mouse atlas from Allen Institute^66^. The region-to-region correlations were also calculated to derive the correlation matrix, which can be visualized using circular graphs. The global functional connectivity strength was calculated by averaging the coefficients over all pairwise region-to-region correlations.

### Logistic regression and bootstrap aggregating algorithm for phenotype prediction

We used the bootstrap aggregating algorithm^28^ to build a classification model for differentiating control, MPS I, and MPS I with AAV-IDUA treatment using multi-resolution correlation maps. We first segmented the 3D support of the seed-based correlation maps into cubic grids of different sizes, 3 × 2 × 1, 6 × 4 × 2, and 12 × 8 × 4 image voxels. Under each segmentation, mean correlations were obtained for each cubic sub-region by averaging the seed-based correlations across the voxels within the cube. These multi-resolution means of correlations were then used as candidate predictors for classifying different mouse groups. We then applied the Bootstrap procedure by randomly sub-sampling 1000 sub-datasets of size 36, which is equal to 80% of the total sample size. Classification models were obtained for each bootstrap sample using the logistic LASSO regression^29^ and the final ensemble network classifier was finally obtained by averaging the 1000 classification models. All analyses in this section were performed using R Statistical Software (v4.2.1; R Core Team 2022).

### Assays for iduronidase and glycoaminoglycans

IDUA activities were determined by a fluorometric assay using 4-methylumbelliferyl alpha-l-iduronide (Glycosynth, #44076) as the substrate as previously described^39^. Glycosaminoglycan levels were determined with the Blyscan Sulfated Glycosaminoglycan Assay (Biocolor, Carrickfergus, UK) as previously described^39^.

### Statistical analyses

Statistical comparisons of IDUA level, GAG level, and global functional connectivity strength within the brain were performed using one-way analysis of variance (ANOVA) or nonparametric Kruskal–Wallis test when normality and equal variance assumptions were violated with post hoc pairwise comparisons with p < 0.05 considered as statistically significant. Group level analyses were conducted on fMRI resting state networks: (1) one-sample t-test was performed on each cohort to obtain group averaged seed maps with p-value < 0.05 (uncorrected); (2) two-sample *t*-test to compare differences between cohorts; (3) two-sample *t*-test to compare the gender differences in each cohort. Two-sample *t*-tests were also applied for each of the predictive locations in the ensemble network classifiers. All statistical analyses were performed using either R Statistical Software (v4.2.1; R Core Team 2022) or MATLAB (The Mathworks Inc., Natick, MA, USA).

### Supplementary Information

Supplementary Figures.

## Data Availability

All data, code, and materials used in the analysis will be available as requested. Please contact Drs. Wei Zhu and Wei Chen for more details.
