# Supplementary material for: Mapping brain networks in MPS I mice and their restoration following gene therapy
Source: Sci Rep. 2023 Aug 5;13:12716. doi: 10.1038/s41598-023-39939-0 (PMC10404260; doi:10.1038/s41598-023-39939-0)
Supplement: Supplementary file 1 — Supplementary Figures. [file 41598_2023_39939_MOESM1_ESM.docx]

Mapping brain networks in MPS I mice and their restoration following gene therapy

**Authors:** Wei Zhu^1,6^, Li Ou^2†^, Lin Zhang^3^, Isaac H. Clark^4^, Ying Zhang^5^, Xiao-Hong Zhu^1,6^, Chester B. Whitley^2^, Perry B. Hackett^7,9^, Walter C. Low^4,8,9,10*^, Wei Chen^1,4,6,9*^

**Affiliations:**

^1^Center for Magnetic Resonance Research, University of Minnesota; Minneapolis, MN 55455, US.

^2^Dept. of Pediatrics, University of Minnesota; Minneapolis, MN 55455, US.

^3^Division of Biostatistics, University of Minnesota; Minneapolis, MN 55455, US.

^4^Biomedical Engineering Graduate Program, University of Minnesota; Minneapolis, MN 55455, US.

^5^Minnesota Supercomputing Institute, University of Minnesota; Minneapolis, MN 55455, US.

^6^Dept. of Radiology, University of Minnesota; Minneapolis, MN 55455, US.

^7^Dept. of Genetics, Cell Biology Development, University of Minnesota; Minneapolis, MN 55455, US.

^8^Dept. of Neurosurgery, University of Minnesota; Minneapolis, MN 55455, US.

^9^Stem Cell Institute, University of Minnesota; Minneapolis, MN 55455, US.

^10^Graduate Program in Neuroscience, University of Minnesota; Minneapolis, MN 55455, US

^†^Current address: Genemagic Biosciences, Media, Pennsylvania, 19063

*Corresponding co-senior authors. Emails: lowwalt@umn.edu and chenx075@umn.edu

**Supplementary Material**


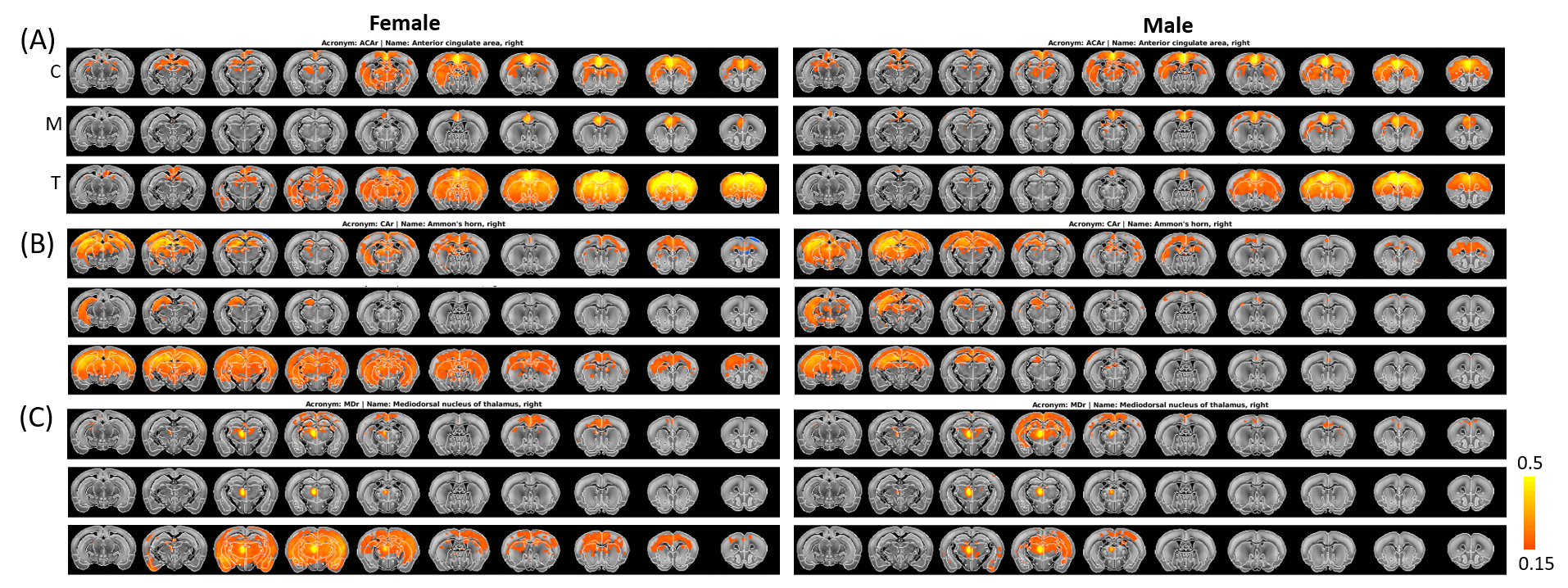


**Fig. S1 Comparison of resting state networks among the three cohorts (C-control, M-mutant, T-treated) between female (left panel) and male (right panel) mice on example seeding reference areas: (A) anterior cingulate cortex of the right-hemispheric brain (ACAr), (B) Ammon's horn in the right-hemispheric brain (CAr), and (C) mediodorsal nucleus of thalamus, right-hemispheric brain (MDr).** The resting state networks were displayed by setting the cross correlation coefficient threshold at 0.15 with p-value < 0.05 (uncorrected).


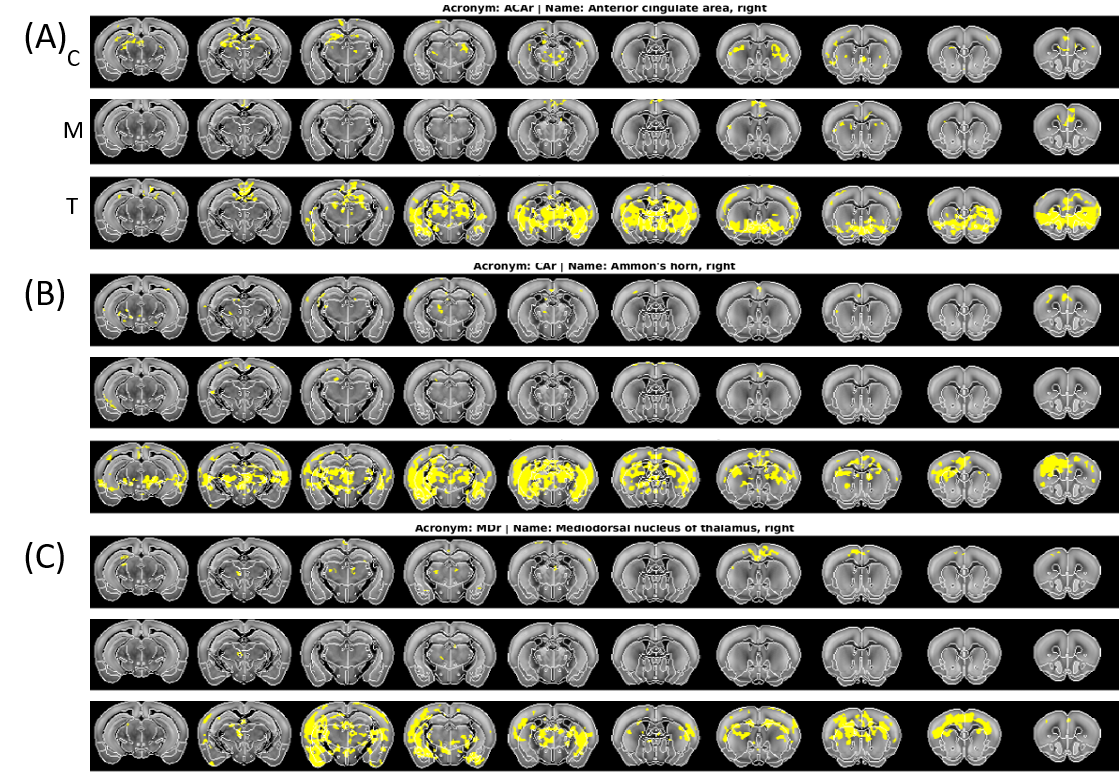


**Fig. S2 Gender differences in RSNs of the three cohorts (C-control, M-mutant and T-treated MPS I mice, see Fig. S1) with example seeding reference areas: (A) anterior cingulate cortex of the right-hemispheric brain (ACAr), (B) Ammon's horn in the right-hemispheric brain (CAr), and (C) mediodorsal nucleus of thalamus, right-hemispheric brain (MDr).** Colored areas have p-values < 0.01 after a two-sample t-test. Note that the large brain areas in the treated cohort indicate a great gender difference in terms of functional connectivity recovery after the gene therapy.


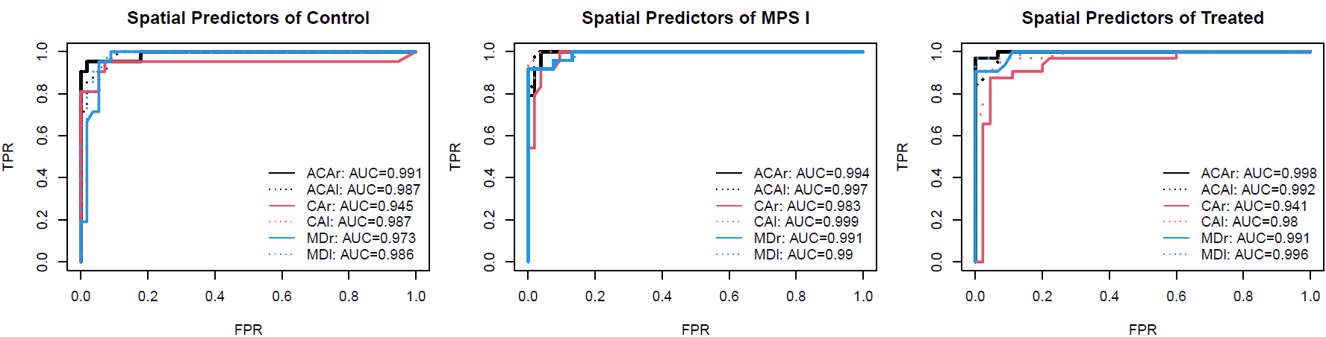


**Fig. S3 Receiver operating curve analysis of AUC accuracy for predicting control, MPS I, and treated MPS I mice using a logistic regression model for ensemble classifiers of ACA, CA, and MD, respectively.**
